# Circulating regulatory T cells predict efficacy and atypical responses in lung cancer patients treated with PD-1/PD-L1 inhibitors

**DOI:** 10.1007/s00262-021-03018-y

**Published:** 2021-07-18

**Authors:** Da Hyun Kang, Chaeuk Chung, Pureum Sun, Da Hye Lee, Song-I Lee, Dongil Park, Jeong Suk Koh, Yoonjoo Kim, Hyon-Seung Yi, Jeong Eun Lee

**Affiliations:** 1 Department of Internal Medicine, College of Medicine, Chungnam National University, 282 Munhwa-ro, Jung-gu, Daejeon, 35015 Korea; 2 College of Medicine, Research Institute for Medical Sciences, Chungnam National University, Daejeon, Korea

**Keywords:** Hyperprogression, Immunotherapy, Lung cancer, PD-1/PD-L1 inhibitor, Pseudoprogression, Regulatory T cell

## Abstract

**Background:**

Immune checkpoint inhibitors (ICIs) have become the standard of care for a variety of cancers, including non-small cell lung cancer (NSCLC). In this study, we investigated the frequency of pseudoprogression and hyperprogression in lung cancer patients treated with ICIs in the real world and aimed to discover a novel candidate marker to distinguish pseudoprogression from hyperprogression soon after ICI treatment.

**Methods:**

This study included 74 patients with advanced NSCLC who were treated with PD-1/PD-L1 inhibitors at Chungnam National University Hospital (CNUH) between January 2018 and August 2020. Chest X-rays were examined on day 7 after the first ICI dose to identify changes in the primary mass, and the response was assessed by computed tomography (CT). We evaluated circulating regulatory T (Treg) cells using flow cytometry and correlated the findings with clinical outcomes.

**Results:**

The incidence of pseudoprogression was 13.5%, and that of hyperprogression was 8.1%. On day 7 after initiation of treatment, the frequency of CD4^+^CD25^+^CD127^lo^FoxP3^+^ Treg cells was significantly decreased compared with baseline (*P* = 0.038) in patients who experienced pseudoprogression and significantly increased compared with baseline (*P* = 0.024) in patients who experienced hyperprogression. In the responder group, the frequencies of CD4^+^CD25^+^CD127^lo^FoxP3^+^ Treg cells and PD-1^+^CD4^+^CD25^+^CD127^lo^FoxP3^+^ Treg cells were significantly decreased 7 days after commencement of treatment compared with baseline (*P* = 0.034 and *P* < 0.001, respectively).

**Conclusion:**

Circulating Treg cells represent a promising potential dynamic biomarker to predict efficacy and differentiate atypical responses, including pseudoprogression and hyperprogression, after immunotherapy in patients with NSCLC.

**Supplementary Information:**

The online version contains supplementary material available at 10.1007/s00262-021-03018-y.

## Introduction

Immune checkpoint inhibitors (ICIs) have transformed cancer treatment to become the standard of care for a variety of cancers, including non-small cell lung cancer (NSCLC). ICIs have shown better clinical efficacy and less toxicity than cytotoxic chemotherapy. Durable clinical responses have been achieved in some patients treated with ICIs; however, their therapeutic efficacy remains limited to 15–30% of treated cancer patients. Only a small minority of patients demonstrate long-term durable responses [1–4], and there is no definite biomarker that can predict ICI efficacy [5].

The management of patients receiving ICIs is challenging due to unpredictable atypical responses, including pseudoprogression and hyperprogression. Pseudoprogression is defined as a transient increase in the size of existing tumor lesions or the appearance of new lesions, followed by subsequent reduction in the tumor burden [6, 7]. This phenomenon is thought to be caused by an immune-related response to ICI treatment, leading to a transient increase in tumor size on imaging [8]. In contrast to expectations after ICI treatment, some patients have experienced rapid and dramatic disease progression following the initiation of ICI treatment, called hyperprogression. Hyperprogression reflects true disease acceleration and leads to adverse outcomes [9–14]. The clinical course of pseudoprogression and hyperprogression varies, but they generally occur early in ICI administration. A clear method of discriminating between pseudoprogression and hyperprogression has not been developed for lesions showing an increase in size on imaging examinations soon after ICI treatment [15, 16].

Regulatory T (Treg) cells contribute to immune suppression by inhibiting the immune response against cancer cells in malignant disease [17, 18]. In patients with early stage NSCLC, increased levels of circulating and tumor-infiltrating Treg cells are correlated with a worse prognosis and a higher risk of recurrence [19, 20]. Several studies have examined the potential of Treg cells as a predictive marker for ICI efficacy in cancer patients. The ratio of peripheral Treg cells to polymorphonuclear myeloid-derived suppressor cells (PMN–MDSCs) has been reported to predict the early response to anti-PD-1 treatment in patients with NSCLC [21]. The presence of actively proliferating PD-1^+^ effector Treg cells in tumors has been reported to be a reliable marker for hyperprogression in gastric cancer patients [22]. A decline in PD-1^+^ circulating Treg cells predicts favorable clinical outcomes in malignant melanoma patients treated with ICIs [23].

In this study, we explored circulating Treg cell subsets as predictive markers of the efficacy of PD-1/PD-L1 inhibitors in patients with advanced NSCLC. We also investigated the frequencies of pseudoprogression and hyperprogression in lung cancer patients treated with ICIs in the real world and aimed to discover a novel candidate marker that can distinguish pseudoprogression from hyperprogression soon after ICI treatment.

## Materials and methods

### Patients and sample collection

This study included patients with advanced NSCLC who were treated with PD-1/PD-L1 inhibitors as monotherapy at Chungnam National University Hospital (CNUH) between January 2018 and August 2020. Patients were given intravenous nivolumab (3 mg/kg body weight every 2 weeks), pembrolizumab (2 mg/kg of body weight or 200 mg every 3 weeks), or atezolizumab (1200 mg every 3 weeks). Treatment was continued until the patient experienced serious adverse events (AEs), had confirmed investigator-assessed disease progression, or withdrew informed consent. Patients expected to experience clinical benefit were permitted to continue treatment beyond radiologic disease progression.

Peripheral blood was collected from patients before treatment (day 0) and 7 days after they received the first dose of PD-1/PD-L1 inhibitor. Peripheral blood mononuclear cells (PBMCs) were isolated from whole blood using standard Ficoll-Paque (GE Healthcare) density gradient centrifugation.

This study was conducted in accordance with the Declaration of Helsinki and Good Clinical Practice guidelines and was approved by the institutional review board of each institution (2018-04-014 at CNUH). All patients were required to provide written informed consent before participating in this study.

### Evaluation

Patient chest X-rays were examined on day 0 and day 7 after the first ICI dose to identify changes in the primary mass and any other measurable lesions. Very small lesions or mediastinal lymph nodes that could not be measured on chest X-ray were excluded from the response evaluation. A response assessment with computed tomography (CT) was performed every three cycles for patients treated with pembrolizumab or atezolizumab, and every four cycles for patients treated with nivolumab. The response to ICI treatment was assessed based on the response evaluation criteria in solid tumors (RECIST), version 1.1. The first CT evaluation of the treatment response was compared with the baseline CT evaluation before immunotherapy. The response was classified as partial response (PR), stable disease (SD), or progressive disease (PD) according to the best overall response to immunotherapy. Responders were defined as patients with complete or partial response or stable disease after treatment with ICI. Durable response was defined as a continuous (complete or partial) objective response lasting at least 6 months after treatment. Tumor growth kinetics (TGK) were defined as the difference in the sum of the largest target lesion diameters according to the RECIST per unit time between the two imaging assessments [24–26]. The TGK ratio was defined as the ratio of TGKpost (after starting immunotherapy) to TGKpre (before starting immunotherapy), and hyperprogression was defined as a TGK ratio ≥ 2 [26]. Pseudoprogression was defined as the confirmation of response after continued treatment when disease progression was shown on a chest X-ray or CT.

Immune-related AEs (irAEs) were defined as dysimmune toxicities caused by immune system imbalance; these toxicities mainly involved the skin, gut, liver, endocrine glands, or lungs, but could affect any tissue. AEs were graded according to the National Cancer Institute Common Terminology Criteria for Adverse Events, version 4.0. Grade 3 or higher irAEs were defined as severe irAEs.

### Multi-color flow cytometry

Multi-color flow cytometry of PBMCs was performed at CNUH. The following human antibodies were used for multi-color flow cytometry: Brilliant Violet 421-conjugated anti-CD25, PE-Cy7 conjugated anti-CD45RA, and APC-Cy7 conjugated anti-CD3 (all from BD Biosciences, San Jose, CA, USA) and Brilliant Violet 605-conjugated anti-CD8, PerCP-Cy5.5-conjugated anti-CD4, FITC-conjugated anti-PD-1, and APC-conjugated anti-CD127 (all from BioLegend, San Diego, CA, USA). For intracellular staining of FOXP3, cells were fixed and permeabilized after surface staining using the Foxp3/Transcription factor staining buffer set (eBioscience) and incubated with PE-conjugated FOXP3 antibody (eBioscience, San Diego, CA, USA). To exclude dead cells, single-cell suspensions were first incubated for 20 min in viability dye (LIVE/DEAD Fixable Aqua, Thermo Fisher). Stained cells were analyzed using BD LSR Fortessa X-20 flow cytometry (BD Biosciences). Fluorescence-activated cell sorting (FACS) analysis was performed using FlowJo software (Tree Star, Ashland, OR, USA).

### PD-L1 expression

PD-L1 expression was assessed using the PD-L1 IHC 22C3 pharmDx test (Agilent Technologies, Santa Clara, CA, USA) on a Dako Autostainer (Dako, Carpinteria, CA, USA) and the PD-L1 IHC SP263 test on the Ventana BenchMark platform (Ventana Medical Systems, Tucson, AZ, USA) in formalin-fixed, paraffin-embedded tumor tissue. The percentage of tumor cells was quantified according to the manufacturers’ recommendations. PD-L1 protein expression was determined based on the percentage of viable tumor cells showing partial or complete membrane staining, defined as the tumor proportion score (TPS) [27]. PD-L1 expression was classified as high if ≥ 50% of the tumor cells stained positive, low if 1–49% of the tumor cells stained positive, and no expression if < 1% of the tumor cells stained positive. Subgroup classification according to PD-L1 expression was performed based on the results of the 22C3 pharmDx assay, and patients without 22C3 pharmDx assay results were classified based on the results of the SP263 assay.

### Statistical analysis

Statistical comparisons were performed using the two-tailed chi-square test and paired t test, with *P* < 0.05 taken to indicate statistical significance. All statistical analyses were performed using Prism, version 6.0 (GraphPad Software, San Diego, CA, USA) and SPSS, version 22 (IBM Corp., Armonk, NY, USA).

## Results

### Patient baseline characteristics

In total, 74 patients were enrolled in this study between January 2018 and August 2020. The baseline characteristics and efficacy outcomes of ICI treatment are summarized in Table 1. The mean age was 69.1 ± 8.8 years, and most patients were male and former/current smokers. The major histological types were squamous cell carcinoma (52.7%) and adenocarcinoma (40.5%). Most patients had stage IV NSCLC and had received at least one previous systemic treatment. A total of 50.0% (37/74) of patients had no/low expression of PD-L1, and 50.0% (37/74) of patients had high PD-L1 expression. Observed best responses were PR in 13 patients (17.6%), SD in 25 patients (33.8%), and PD in 36 patients (48.6%). Any grade irAEs occurred in 21 patients (28.4%), and severe irAEs occurred in 15 patients (20.3%).

**Table 1:** Baseline characteristics and efficacy outcomes of all patients (N = 74)

Variable		Mean ± standard deviation or number of patients (%)
Age, years		69.1 ± 8.8
Sex	Male	61 (82.4)
	Female	13 (17.6)
Smoking status	Never	14 (18.9)
	Former/current	60 (81.1)
Histology	Adenocarcinoma	30 (40.5)
	Squamous	39 (52.7)
	Other*	5 (6.8)
EGFR	Wild-type	74 (100.0)
	Mutant	0 (0.0)
ALK rearrangement	Negative	73 (98.6)
	Positive	1 (1.4)
PD-L1 expression**	No (TPS < 1%)	20 (27.1)
	Low (TPS 1–49%)	17 (23.0)
	High (TPS ≥ 50%)	37 (50.0)
Number of prior regimens	0	9 (12.2)
	1	54 (73.0)
	≥ 2	11 (14.9)
Agent	Nivolumab	16 (21.6)
	Pembrolizumab	31 (41.9)
	Atezolizumab	27 (36.5)
Best response to treatment	PR	13 (17.6)
	SD	25 (33.8)
	PD	36 (48.6)
PseudoPD/HyperPD	Pseudoprogression	10 (13.5)
	Hyperprogression	6 (8.1)
Immune-related AEs	Any grade	21 (28.4)
	Severe AEs	15 (20.3)

Abbreviations: *EGFR* epidermal growth factor receptor, *ALK* anaplastic lymphoma kinase, *PD-L1* programmed death-ligand 1, *TPS* tumor proportion score, *PR* partial response, *SD* stable disease, *PD* progression disease, *PseudoPD* pseudoprogression, *HyperPD* hyperprogression, *AEs*, adverse events

*One adenosquamous, one large cell, three non-small cell lung cancer not otherwise specified

**Subgroup classification according to PD-L1 expression was based on the results of the 22C3 pharmDx assay. Patients without 22C3 pharmDx assay results were classified based on the results of the SP263 assay

### Radiologic evaluation

**Fig. 1:**
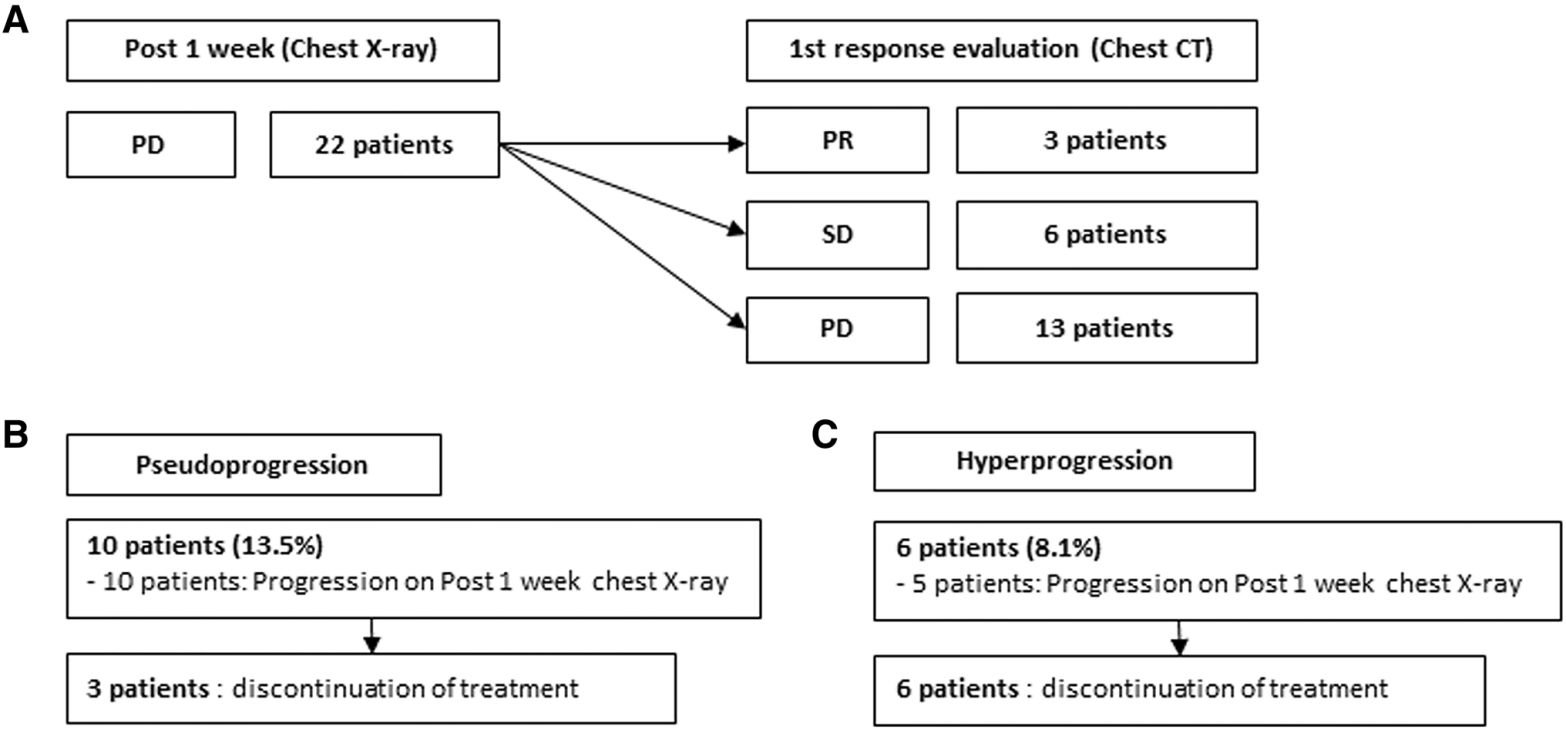
Response evaluation by chest X-ray at 1 week post-treatment and chest computed tomography (CT) after 3–4 cycles of treatment in lung cancer patients treated with PD-1/PD-L1 immune checkpoint inhibitors (ICIs). **a** In 22 patients identified as showing progressive disease (PD) on chest X-rays at 7 days post-treatment, three patients showed a partial response (PR), six showed stable disease (SD), and 13 showed PD on a first CT response evaluation that was performed at 2 months after ICI administration. **b** All ten patients who experienced pseudoprogression showed progression on chest X-rays at 7 days post-treatment, among which nine showed PR or SD on the first CT response evaluation. Only one patient showed PD on the first CT response evaluation, followed by PR on the second CT. Among patients with pseudoprogression, three discontinued treatment. **c** Among the six patients who experienced hyperprogression, five showed progression on chest X-rays at 7 days post-treatment, and all patients discontinued treatment

The response to treatment according to chest X-rays on day 7 after initiation of ICI treatment was PR in one patient (1.4%), SD in 51 patients (68.9%), and PD in 22 patients (29.7%). Among the 22 patients with PD on day 7, 3 had PR, 6 had SD, and 13 had PD according to the first CT evaluation of response, which was performed at 2 months after administration of the first dose of ICI (Fig. 1a). The incidence of pseudoprogression was 13.5% and that of hyperprogression was 8.1%. All 10 patients who experienced pseudoprogression showed progression on chest X-rays on day 7 after initiation of treatment, among which nine showed PR or SD on the first CT response evaluation. Only one patient showed PD on the first CT response evaluation, followed by PR on the secondary CT response evaluation. Among patients with pseudoprogression, three discontinued treatment (Fig. 1b). Of the six patients who experienced hyperprogression, five showed progression on chest X-rays at 7 days post-treatment, and all patients discontinued treatment (Fig. 1c).

### Circulating regulatory T cells in pseudoprogression and hyperprogression

**Fig. 2:**
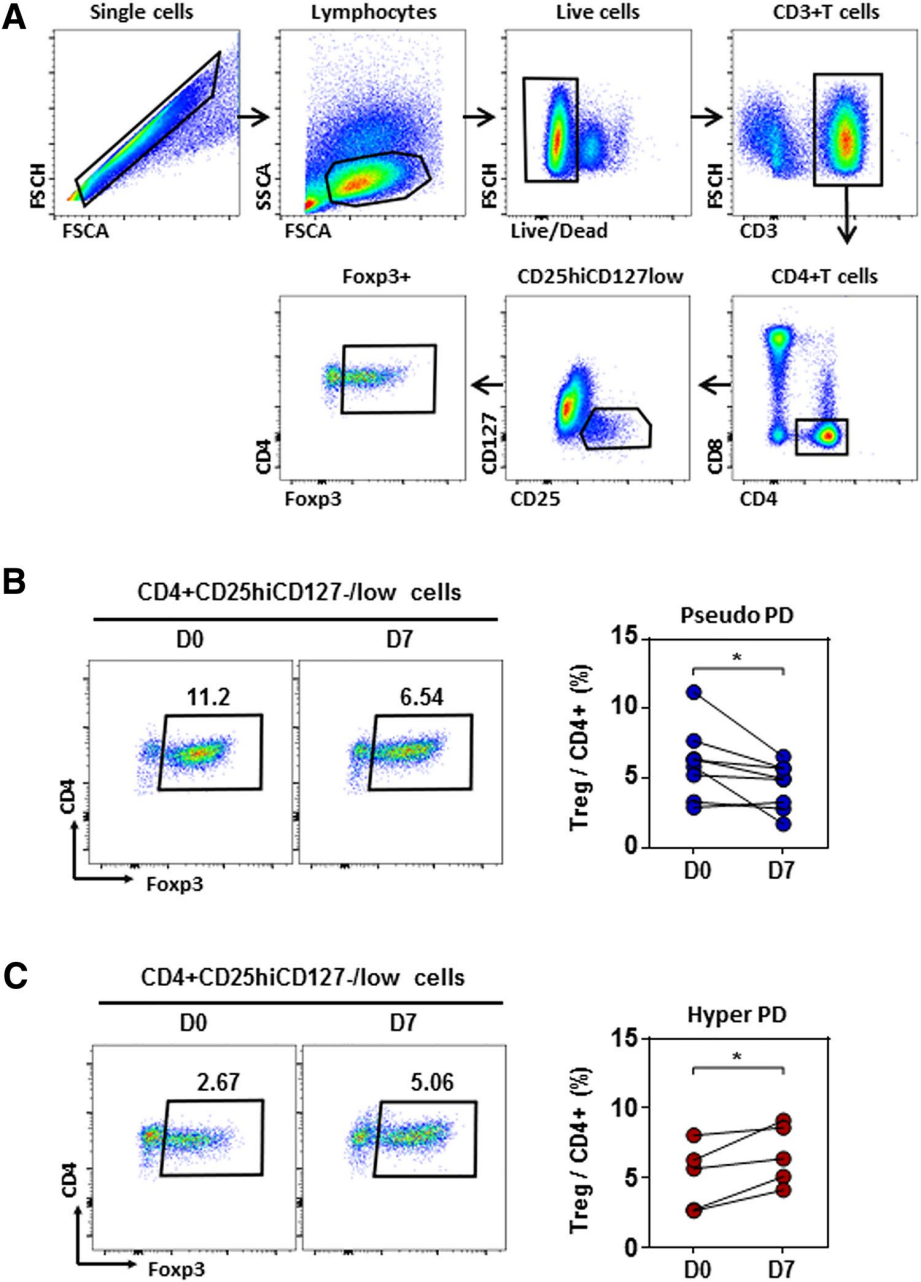
Circulating regulatory T (Treg) cells in pseudoprogression and hyperprogression. **a** Gating strategies of CD4^+^CD25^+^CD127^lo^FoxP3^+^ Treg cells by flow cytometry. **b** In patients who experienced pseudoprogression, the frequency of CD4^+^CD25^+^CD127^lo^FoxP3^+^ Treg cells decreased significantly 7 days after initiation of treatment compared with baseline (*P* = 0.038). **c** In patients who experienced hyperprogression, the frequency of CD4^+^CD25^+^CD127^lo^FoxP3^+^ Treg cells increased significantly 7 days after initiation of treatment compared with baseline (*P* = 0.024)

We examined the relative frequencies and phenotypes of subpopulations of peripheral blood CD4^+^ T cells at baseline and at 7 days post-treatment by evaluating the relative frequency of CD4^+^CD25^+^CD127^lo^FoxP3^+^ Treg cells. The gating strategies applied in flow cytometric analyses are shown in Fig. 2a. In patients who experienced pseudoprogression, the frequency of CD4^+^CD25^+^CD127^lo^FoxP3^+^ Treg cells was significantly decreased on day 7 after initiation of treatment compared with baseline (*P* = 0.038, Fig. 2b), whereas in patients who experienced hyperprogression, the frequency of CD4^+^CD25^+^CD127^lo^FoxP3^+^ Treg cells was significantly increased on day 7 compared with baseline (*P* = 0.024, Fig. 2c). The clinical baseline characteristics and changes in the relative frequency of CD4^+^CD25^+^CD127^lo^FoxP3^+^ Treg cells in patients with pseudoprogression and hyperprogression are shown in Supplementary Tables 1 and 2.

### Clinical outcomes according to circulating regulatory T cells

**Fig. 3:**
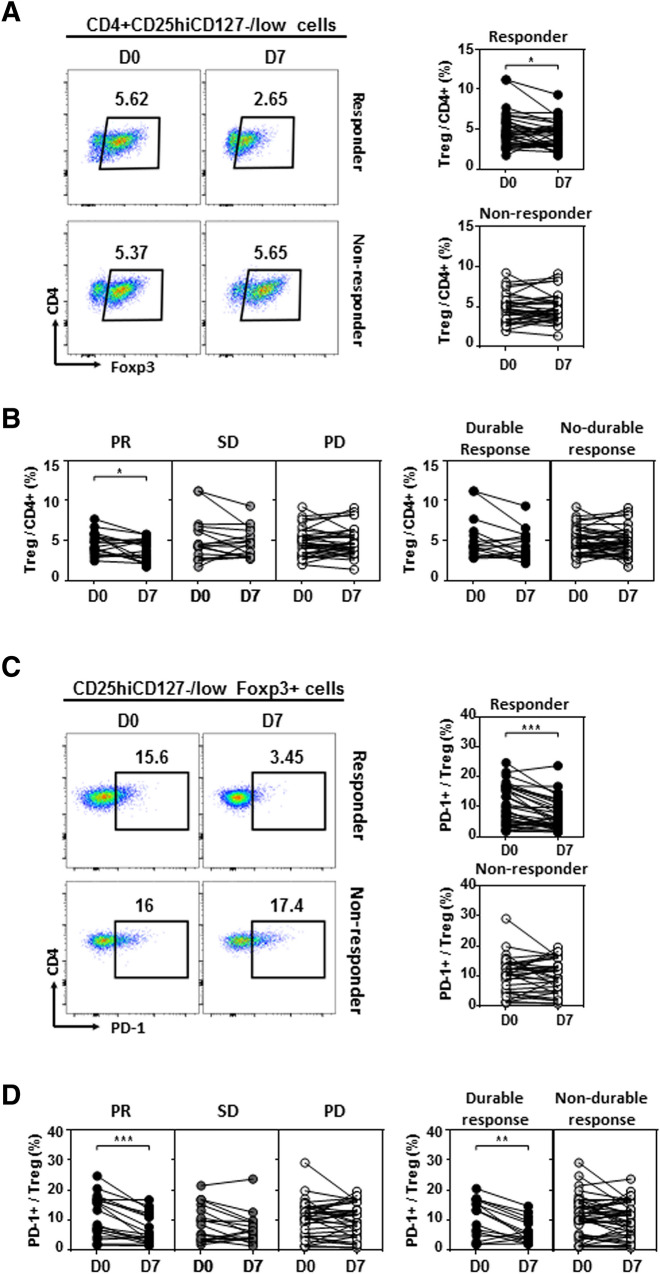
Changes in circulating Treg cells according to clinical outcomes. **a** In the responder group, the frequency of CD4^+^CD25^+^CD127^lo^FoxP3^+^ Treg cells decreased significantly 7 days after initiation of treatment compared with baseline (*P* = 0.034). **b** The frequency of CD4^+^CD25^+^CD127^lo^FoxP3^+^ Treg cells decreased significantly in patients with a PR (*P* = 0.035), and there was no significant change in patients with SD or PD. **c** In the responder group, the frequency of PD-1^+^CD4^+^CD25^+^CD127^lo^FoxP3^+^ Treg cells decreased significantly 7 days after initiation of treatment compared with baseline (*P* < 0.001). (D) In patients with a PR, the frequency of PD-1^+^CD4^+^CD25^+^CD127^lo^FoxP3^+^ Treg cells decreased significantly (*P* < 0.001), and a significant decline was also observed in patients who showed a durable response (*P* = 0.002)

We evaluated the relative frequencies of CD4^+^CD25^+^CD127^lo^FoxP3^+^ Treg cells and PD-1^+^CD4^+^CD25^+^CD127^lo^FoxP3^+^ Treg cells according to clinical outcomes. In the responder group, the frequency of CD4^+^CD25^+^CD127^lo^FoxP3^+^ Treg cells was significantly decreased on day 7 after initiation of treatment compared with baseline (*P* = 0.034, Fig. 3a). In particular, the frequency of CD4^+^CD25^+^CD127^lo^FoxP3^+^ Treg cells was significantly decreased in patients with a PR (*P* = 0.035), and there was no significant change in patients with SD or PD (Fig. 3b). In the responder group, the frequency of PD-1^+^CD4^+^CD25^+^CD127^lo^FoxP3^+^ Treg cells was significantly decreased on day 7 after initiation of treatment compared with baseline (*P* < 0.001, Fig. 3c). In patients with a PR, the frequency of PD-1^+^CD4^+^CD25^+^CD127^lo^FoxP3^+^ Treg cells was significantly decreased (*P* < 0.001), and a significant decline also occurred in patients who showed a durable response (*P* = 0.002, Fig. 3d).

Among the 74 patients, we paired baseline and 7 days post-treatment samples for 68 patients. Among these, 30 patients showed increased frequency of circulating CD4^+^CD25^+^CD127^lo^FoxP3^+^ Treg cells on day 7 after initiation of treatment, and 38 patients showed a decrease. The disease control rate of patients with decreased circulating CD4^+^CD25^+^CD127^lo^FoxP3^+^ Treg cells was 63.2%, which was significantly higher than the rate of 36.7% in patients with increased circulating CD4^+^CD25^+^CD127^lo^FoxP3^+^ Treg cells. The frequency of hyperprogression was 16.7% in patients with increased circulating Treg cells, which was significantly higher than 0.0% in patients with decreased circulating Treg cells (*P* = 0.009). The incidence of pseudoprogression was 3.3% in patients with increased circulating Treg cells, versus 18.4% in patients with decreased circulating Treg cells (*P* = 0.055, Table 2).

**Table 2:** Clinical outcomes according to changes in circulating regulatory T cells

Variable		Patients with increased circulating regulatory T cells (*N* = 30)	Patients with decreased circulating regulatory T cells (*N* = 38)	*P* value
Response	PR	4 (13.3)	9 (23.7)	
	SD	7 (23.3)	15 (39.5)	0.095
	PD	19 (63.3)	14 (36.8)	
Disease control rate		36.7%	63.2%	0.030
PseudoPD/HyperPD	Pseudoprogression	1 (3.3)	7 (18.4)	0.055
	Hyperprogression	5 (16.7)	0 (0.0)	0.009
Immune-related AEs	Any grade	11 (36.7)	10 (26.3)	0.359
	Severe AEs	8 (26.7)	7 (18.4)	0.416

Abbreviations: *PR* partial response, *SD* stable disease, *PD* progression disease, *PseudoPD* pseudoprogression, *HyperPD* hyperprogression, *AEs* adverse events

Among 68 patients with pre- and post-treatment paired samples, 26 patients showed an increase in circulating PD-1^+^CD4^+^CD25^+^CD127^lo^FoxP3^+^ Treg cells, and 42 patients showed a decrease. The best response to treatment differed significantly between the two groups (*P* = 0.021), and the disease control rate among patients with decreased circulating PD-1^+^CD4^+^CD25^+^CD127^lo^FoxP3^+^ Treg cells was 64.3%, which was significantly higher than the rate of 30.8% in patients with increased circulating PD-1^+^ Treg cells (*P* = 0.007). The incidence of hyperprogression in patients with increased circulating PD-1^+^CD4^+^CD25^+^CD127^lo^FoxP3^+^ Treg cells was 15.4%, which was significantly higher than that of 2.4% in patients with decreased circulating PD-1^+^ Treg cells (*P* = 0.046). There was no significant difference in immune-related AE incidence between the two groups (Table 3).

**Table 3:** Clinical outcomes according to changes in circulating PD-1 + regulatory T cells

Variable		Patients with increased circulating PD-1 + regulatory T cells (N = 26)	Patients with decreased circulating PD-1 + regulatory T cells (N = 42)	*P* value
Response	PR	2 (7.7)	11 (26.2)	0.021
	SD	6 (23.1)	16 (38.1)	
	PD	18 (69.2)	15 (35.7)	
Disease control rate		30.8%	64.3%	0.007
PseudoPD/HyperPD	Pseudoprogression	1 (3.8)	7 (16.7)	0.111
	Hyperprogression	4 (15.4)	1 (2.4)	0.046
Immune-related AEs	Any grade	8 (30.8)	13 (31.0)	0.987
	Severe AEs	7 (26.9)	8 (19.0)	0.447

Abbreviations: *PR* partial response, *SD* stable disease, *PD* progression disease, *PseudoPD* pseudoprogression, *HyperPD* hyperprogression, *AEs* adverse events

## Discussion

This was the first study to demonstrate that the frequency of circulating CD4^+^CD25^+^CD127^lo^FoxP3^+^ Treg cells has potential as a dynamic marker for distinguishing pseudoprogression from hyperprogression and for predicting the response to ICI treatment in advanced NSCLC patients. It is very important to discriminate between psuedoprogression, hyperprogression, and real progression when imaged tumor size increases in the early stage among patients treated with ICIs. Unlike in other cancers, it is very difficult to perform repeated invasive tissue biopsy in lung cancer patients; therefore, a peripheral blood biomarker is needed. Baseline derived neutrophil-to-lymphocyte ratios (dNLR) ≤ 3 or baseline lactate dehydrogenase (LDH) levels more than double the upper limit of normal have been reported as more common in hyperprogression than in standard progression patients [11], but no significant difference was reported in another study [28]. Baseline low highly differentiated CD28^−^CD27^−^CD4 T cells (T_HD_ cells), and increase in the % T_HD_ cells from baseline were present in 100% of patients with hyperprogression, but validation of this finding using larger samples is required [29]. There is limited evidence of an association between peripheral blood markers and pseudoprogression [16]. In melanoma patients, LDH levels below the upper limit of normal at baseline were reported to be associated with pseudoprogression [30], but it is difficult to regard LDH as a predictive marker for pseudoprogression, since other studies have reported no correlation [16]. To date, no definite markers have been identified for discrimination between hyperprogression and pseudoprogression.

Pseudoprogression is commonly defined as an increase in tumor size or the appearance of new lesions, followed by tumor reduction or maintenance of stable disease over the course of ICI treatment [24]. Pseudoprogression is rare among NSCLC patients treated with PD-1 inhibitors, with reported incidence rates from 0.8 to 2% [7, 31, 32]. Pseudoprogression has been reported to occur mainly 2–8 weeks after ICI treatment. However, based on a report that proliferative response in the peripheral blood reached a peak in the first week in response to a PD-1 blocker, an immune response such as pseudoprogression may appear earlier than might be expected [33]. In this study, pseudoprogression was found in 10 patients (13.5%), which is a higher frequency than previously reported, perhaps because patients whose tumor size temporarily increased according to chest X-rays at 7 days post-treatment were also included in pseudoprogression. Since most of these patients were identified as showing PR or SD on the primary response evaluation CT, it is highly likely that early onset pseudoprogression was not included in the pseudoprogression reported in several previous studies. Thus, chest X-ray deterioration at the initial stage of treatment is important despite pseudoprogression, since various symptoms are caused by increased tumor size, causing cessation of immunotherapy in 3 of 10 patients in this study. Of the three patients who discontinued treatment, two discontinued immunotherapy temporarily and received steroid treatment when symptomatic pseudoprogression occurred. During steroid treatment, the patients’ general condition worsened, eventually rendering retreatment with ICIs impossible. One patient maintained a PR, as determined by chest CT, after pseudoprogression, but drug eruption occurred as an irAE due to PD-1 blocker, and the drug was discontinued. Several studies have also reported pseudoprogression symptoms such as increased pleural effusion and cardiac tamponade; in such cases, the drug was discontinued in most patients [34, 35]. If clinicians could identify pseudoprogression in the early stage, steroids could be used to relieve severe immune-related reactions, and ICI treatment could continue in anticipation of the treatment effect. In lung cancer patients, the incidence of hyperprogression after ICI treatment varies from 5 to 19.2% [24]. In patients who experienced hyperprogession, increased tumor size was observed in most of the patients by chest X-ray at 7 days post-treatment, when the duration of ICI administration was very short (< 1.5 months). In about half of patients with hyperprogression, ICI treatment was discontinued within 3 weeks after the first dose due to rapid deterioration of tumor lesions. In patients who experience progression within 3 weeks of initial treatment, many clinicians attempt to differentiate among hyperprogression, pseudoprogression, and true progression according to various criteria and factors, but no useful markers have been identified to date. In this study, circulating regulatory T cells were identified as a useful marker for discriminating between pseudoprogression and hyperprogression when chest X-ray evaluation on day 7 after initiation of treatment indicated an increased tumor size.

Regulatory T cells are an immunosuppressive subset of T cells identified as CD4^+^CD25^+^CD127^lo^, characterized by expression of the master regulatory transcription factor, Forkhead box protein P3 (FoxP3). In cancer, regulatory T cells promote tumorigenesis by exerting suppressive activities on effector cells, causing inactivation or apoptosis [36]. Treg cells have been reported to induce tumor resistance against ICIs, leading to tumor relapse and poor prognosis through various signaling pathways including upregulation of other immune checkpoints, Treg-induced TGF-β activation and production, and increased activation of the PI3K signaling pathway [37, 38]. However, most studies of Treg mechanisms have been conducted using tumor-infiltrating lymphocytes (TILs) from the tumor microenvironment, and no studies have investigated the relationship between changes in circulating Treg cells before and after immunotherapy treatment. In this study, we found that changes in circulating Treg cells in the early phase after initiation of ICI treatment could significantly predict clinical benefit and discriminate between pseudoprogression and hyperprogression. Proliferation of PD-1^+^ effector Treg cells has been reported to be a marker for predicting hyperprogression in gastric cancer patients, but only in a study that used Treg cells within tumors [22]. Another study reported that a decline in PD-1^+^ circulating Treg cells was associated with favorable clinical outcomes in malignant melanoma patients receiving ICI treatment, but it was conducted using a small number of patients and 2- or 3-week post-treatment blood collection periods [23], conditions that differed from those used in our study. In this study, early decline of PD-1^+^ circulating Treg cells was associated with favorable outcome for ICI, and early increase predicted hyperprogression. In a real clinical setting, it is crucial to discriminate between pseudoprogression and hyperprogression in the early stages; therefore, circulating Treg cells and PD-1^+^ Treg cells would be useful as early dynamic biomarkers.

This study has some limitations. First, the basic mechanism underlying the correlation between circulating Treg cells and treatment response has not been established. Further experiments are needed to elucidate the mechanism by which an increase in circulating Treg cells is associated with hyperprogression and a decrease in Treg cells is associated with clinical benefit and pseudoprogression. Second, since the frequency of either pseudoprogression or hyperprogression is not high, the number of patients involved in this study was insufficient to reach a definitive conclusion. Therefore, our results must be further validated in larger cohorts.

In this study, we found that an early change in the frequency of circulating Treg cells was associated with a clinical benefit and can help distinguish between pseudoprogression and hyperprogression when an increase in tumor size is observed on chest X-ray 7 days after commencement of treatment. In conclusion, circulating Treg cells have potential as a dynamic biomarker for predicting efficacy and differentiating atypical responses, including pseudoprogression and hyperprogression, after immunotherapy in patients with NSCLC.

### Supplementary Information

Below is the link to the electronic supplementary material.

Supplementary file1 (DOCX 15 kb)
