# Supplementary material for: Circulating regulatory T cells predict efficacy and atypical responses in lung cancer patients treated with PD-1/PD-L1 inhibitors
Source: Cancer Immunol Immunother. 2021 Jul 18;71(3):579–88. doi: 10.1007/s00262-021-03018-y (PMC8854239; doi:10.1007/s00262-021-03018-y)
Supplement: Supplementary file 1 — Supplementary file1 (DOCX 15 kb) [file 262_2021_3018_MOESM1_ESM.docx]

Supplementary Table 1. Patients who experienced pseudoprogression after immunotherapy.

| Patient  (no.) | Age | Sex | Smoking status | Histology | PD-L1 expression | Agent | Chest X-ray 7 days post-treatment | CT at 2 months post-treatment | Treatment duration (months) | Treg/CD4 at baseline (%) | Treg/CD4 at 7 days post-treatment (%) |
| --- | --- | --- | --- | --- | --- | --- | --- | --- | --- | --- | --- |
| 1 | 72 | M | Current | SqCC | High | Pembrolizumab | PD | PR | 7.3 | 2.88 | 3.25 |
| 2 | 79 | M | Former | SqCC | High | Pembrolizumab | PD | SD | 14.5 | 11.2 | 6.54 |
| 3 | 57 | F | None | Adeno | Low | Nivolumab | PD | SD | 2.3 | – | 6.51 |
| 4 | 75 | M | Current | Adeno | No | Atezolizumab | PD | SD | 8.4 | 4.38 | – |
| 5 | 79 | M | Former | SqCC | High | Pembrolizumab | PD | PR | 3.0 | 3.28 | 2.8 |
| 6 | 63 | M | Former | SqCC | High | Pembrolizumab | PD | SD | 5.6 | 5.82 | 1.69 |
| 7 | 81 | M | Former | SqCC | High | Pembrolizumab | PD | PR | 3.9 | 5.22 | 4.9 |
| 8 | 61 | M | Former | SqCC | Low | Atezolizumab | PD | PD | 2.2 | 6.3 | 5.7 |
| 9 | 55 | M | Current | Adeno | High | Pembrolizumab | PD | SD | 18.0 | 7.69 | 5.71 |
| 10 | 67 | M | Former | SqCC | No | Atezolizumab | PD | SD | 5.1 | 6.38 | 4.94 |

Abbreviations: PD-L1, programmed death-ligand 1; Adeno, adenocarcinoma; SqCC, squamous cell carcinoma; PD, progressive disease; PR, partial response; SD, stable disease.

Supplementary Table 2. Patients who experienced hyperprogression after immunotherapy.

| Patient  (no.) | Age | Sex | Smoking status | Histology | PD-L1 expression | Agent | Chest X-ray 7 days post-treatment | CT 2 months post-treatment | Treatment duration (months) | Treg/CD4 at baseline (%) | Treg/CD4 at 7 days post-treatment (%) |
| --- | --- | --- | --- | --- | --- | --- | --- | --- | --- | --- | --- |
| 1 | 58 | M | Current | Adeno | No | Nivolumab | PD | PD | 0.0 | 5.63 | 6.35 |
| 2 | 69 | F | None | Adeno | Low | Nivolumab | PD | PD | 0.7 | 3.85 | – |
| 3 | 81 | M | Former | Adeno | High | Pembrolizumab | PD | PD | 0.0 | 8.03 | 8.58 |
| 4 | 70 | M | Former | Adeno | No | Nivolumab | PD | PD | 0.0 | 6.25 | 9.1 |
| 5 | 65 | M | Former | SqCC | Low | Atezolizumab | SD | PD | 1.4 | 2.59 | 4.09 |
| 6 | 73 | M | Former | SqCC | Low | Nivolumab | PD | PD | 0.9 | 2.67 | 5.06 |

Abbreviations: PD-L1, programmed death-ligand 1; Adeno, adenocarcinoma; SqCC, squamous cell carcinoma; PD, progressive disease; PR, partial response; SD, stable disease.
